# Influenza returns with a season dominated by clade 3C.2a1b.2a.2 A(H3N2) viruses, WHO European Region, 2021/22

**DOI:** 10.2807/1560-7917.ES.2022.27.15.2200255

**Published:** 2022-04-14

**Authors:** Angeliki Melidou, Csaba Ködmön, Karen Nahapetyan, Annette Kraus, Erik Alm, Cornelia Adlhoch, Piers Mooks, Nishi Dave, Carlos Carvalho, Margaux MI Meslé, Rodney Daniels, Richard Pebody, Members of the WHO European Region influenza surveillance network, Members of the WHO European Region influenza surveillance network that contributed virus characterisation data, Irena Tabain, Amanda Bolt Botnen, Ramona Trebbien, Niina Ikonen, Outi Lyytikäinen, Marianne Wedde, Ralf Dürrwald, Charlene Bennett, Jeff Connell, Simona Puzelli, Marzia Facchini, Adam Meijer, Ron A.M. Fouchier, Maja Vukovikj, Elizabeta Janchevska, Karoline Bragstad, Olav Hungnes, Andrey Komissarov, Kirill Stolyarov, Francisco Pozo, Inmaculada Casas, Ana Rita Gonçalves, Tania Spedaliero, Catherine Moore, Simon Cottrell

**Affiliations:** 1 European Centre for Disease Prevention and Control (ECDC), Stockholm, Sweden; 2 World Health Organization (WHO) Regional Office for Europe, Copenhagen, Denmark; 3 WHO Collaborating Centre, Francis Crick Institute, London, United Kingdom; 4 Members of the WHO European Region influenza surveillance network that contributed virus characterisation data are listed under collaborators

**Keywords:** surveillance, influenza virus, virus characterisation, pandemic, Europe

## Abstract

In the WHO European Region, COVID-19 non-pharmaceutical interventions continued slowing influenza circulation in the 2021/22 season, with reduced characterisation data. A(H3) predominated and, in some countries, co-circulated with A(H1)pdm09 and B/Victoria viruses. No B/Yamagata virus detections were confirmed. Substantial proportions of characterised circulating virus subtypes or lineages differed antigenically from their respective northern hemisphere vaccine components. Appropriate levels of influenza virus characterisations should be maintained until the season end and in future seasons, when surveillance is adapted to integrate SARS-CoV-2.

This study describes the 2021/22 influenza season in the World Health Organization (WHO) European Region through results of influenza virus detection and typing up to week 10 2022. Viral characterisations reported to The European Surveillance System (TESSy) up to week 4 2022 are also presented, taking into consideration continued implications of the coronavirus disease (COVID-19) pandemic. Based on the WHO Collaborating Centre (CC)’s assessments for the 2022 northern hemisphere (NH) vaccine composition meeting (VCM), similarity of circulating influenza viruses to the 2021/22 NH vaccine components are discussed.

## Description of the 2021/22 influenza season up to week 10 2022 

**Figure 1:**
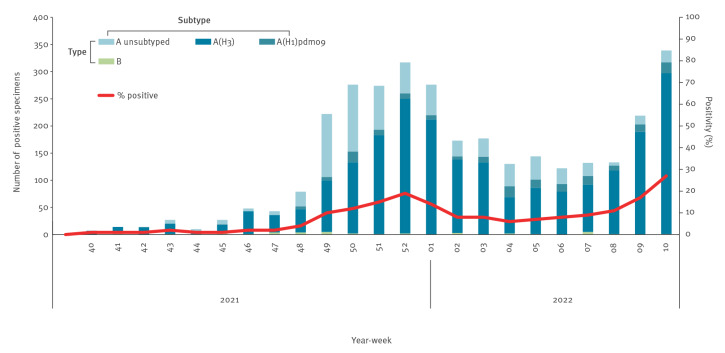
Distribution over time of the numbers of specimens positive for influenza in primary-care-sentinel surveillance (n = 3,083), as well as the proportions of positive specimens among those tested (n = 39,551), WHO European Region, week 40 2021–week 10 2022

After scant influenza virus circulation in 2020/21, the 2021/22 influenza season started in week 49 2021 in the WHO European Region. In week 2 2022, positivity in sentinel primary care specimens dipped below the epidemic threshold of 10% but rose again above it in weeks 8 to 10 2022 (Figure 1). From week 40 2021 to week 10 2022, 55,049 influenza virus detections from sentinel and non-sentinel sources were reported to TESSy by 50 countries and territories of the Region (Supplement 1). Based on sentinel detections, AH1pdm09 predominated in France [1]. In all the other countries, A(H3) was the major influenza A subtype [1], co-circulating with A(H1)pdm09 subtype and to a lesser extent type B viruses.

WHO: World Health Organization.

In the 2020 southern hemisphere (SH) and the 2020/21 NH influenza seasons, circulation of influenza viruses was shown to be very limited [2]. In 2021, severe acute respiratory syndrome coronavirus 2 (SARS-CoV-2)-variant waves dominated by Delta (Phylogenetic Assignment of Named Global Outbreak (Pango) lineage designation:  B.1.617.2) and Omicron (Pango: B.1.1.529) coincided with the course of the 2021/22 influenza season in the European Region [3]. Despite an overall increased testing for influenza viruses from sentinel and non-sentinel sources in the Region, which was probably related to enhanced testing for SARS-CoV-2, the total number of influenza virus detections appeared to remain lower than in seasons before the COVID-19 pandemic as shown in Supplement 1. Public health measures aimed at limiting circulation of SARS-CoV-2 likely could have contributed to this reduced positivity. Although sentinel sources continued reporting during the COVID-19 pandemic, data for the 2021/22 influenza season reflected increased testing in some of these sources, while others either did not test or reported on reduced numbers tested (Supplement 1).

## Influenza virus characterisation 

European influenza national reference laboratories in the Region collect virological influenza surveillance data, perform virus characterisations and report weekly aggregated and strain-based data to TESSy. The Francis Crick Institute, WHO Collaborating Centre (WHO CC) for Reference and Research on Influenza based in London, United Kingdom (UK), provides laboratories with post-infection ferret antisera for antigenic characterisation using haemagglutination inhibition assays. The Francis Crick Institute WHO CC also provides a list of reference sequences for the assignment of viruses to haemagglutinin (HA) gene clades/subclades following sequencing [4,5].

In addition to the Francis Crick Institute WHO CC, there are globally six designated WHO CCs. WHO CCs conduct research and analyses on influenza data or samples, provided by National Influenza Centers (NICs) [6]. Laboratories ship influenza specimens to WHO CCs for further in-depth analyses essential for the WHO Vaccine Composition Meeting (VCM) decisions [7].

## Reporting of viral characterisations to The European Surveillance System in the beginning of the 2021/22 season

**Figure 2:**
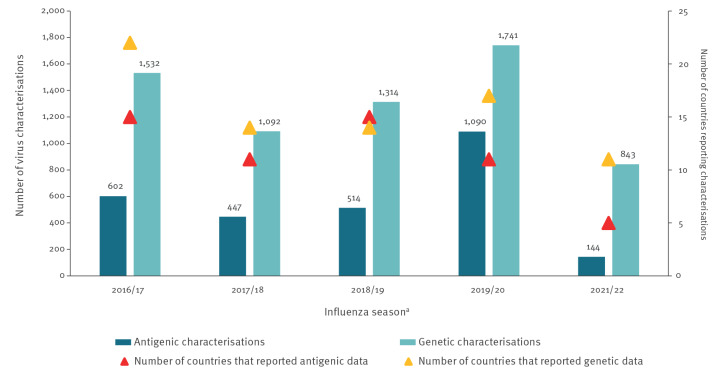
Numbers of antigenic and genetic virus characterisations used for the northern hemisphere Vaccine Composition Meeting decisions in each influenza season^a^ and numbers of countries reporting such characterisations, WHO European Region, 2016/17–2021/22 (n = 9,319 characterisations)^a^

Compared with 2019/20 and 2018/19, seemingly lower amounts of antigenic and genetic characterisation data in 2021/22 were reported to TESSy ahead of the VCM on 25 February 2022 (Figure 2). The WHO European Region Member States reporting characterisations also appeared to be fewer, with the most apparent decrease observed in numbers of countries providing antigenic data. The reason might have been that antigenic characterisation is more laborious. Combinations of reduced human or laboratory resources and biosafety concerns related to the possibility of SARS-CoV-2 co-infection might have also played a role [8].

WHO: World Health Organization.

^a^ Influenza season 2020/21 is not presented because of very low numbers.

Up to week 4 2022, 11 of 50 countries submitted virus genetic characterisation data to TESSy for the 2021/22 season (Table 1). Antigenic characterisation data were reported to TESSy by six countries. Of all viruses detected, < 1% (144/34,653) were characterised antigenically and 2% (843/34,653) genetically [9]. Ideally, all or at least 10% of the viruses originating from sentinel sources should be genetically characterised [10,11].

**Table 1:** Number of viruses characterised antigenically and genetically as reported to TESSy^a^ by country and week of sampling, WHO European Region, week 40 2021–week 4 2022 (n = 987 characterisations)

Reporting country	Characterisation	2021 Week number													2022 Week number				Total
		40	41	42	43	44	45	46	47	48	49	50	51	52	01	02	03	04	
Croatia	AG	0	0	0	0	0	0	0	0	0	0	0	0	0	0	0	0	0	**0**
	GEN	0	0	1	0	0	0	0	0	0	0	0	0	0	0	0	0	0	**1**
Denmark	AG	0	0	0	0	0	0	0	0	0	1	0	1	0	0	0	0	0	**2**
	GEN	1	1	1	4	1	0	3	3	6	18	8	27	19	14	17	9	2	**134**
Finland	AG	0	0	0	0	0	0	0	0	0	0	0	0	0	0	0	0	0	**0**
	GEN	1	0	0	2	0	0	2	3	1	0	0	0	0	0	0	0	0	**9**
Germany	AG	1	1	0	1	1	0	0	2	0	0	2	4	4	8	4	5	1	**34**
	GEN	0	0	0	1	0	0	2	2	0	0	0	0	0	0	0	0	0	**5**
Ireland	AG	0	0	0	0	0	0	0	0	0	0	0	0	0	0	0	0	0	**0**
	GEN	0	0	0	0	0	0	2	0	1	1	0	4	1	1	4	0	0	**14**
Italy	AG	0	0	0	0	0	0	0	0	0	0	0	0	0	0	0	0	0	**0**
	GEN	0	0	0	1	4	1	0	1	0	0	2	1	0	0	0	0	0	**10**
Netherlands	AG	0	0	0	0	0	0	0	0	0	0	0	0	0	0	0	0	0	**0**
	GEN	13	21	30	29	13	16	15	13	12	6	11	18	20	9	9	5	0	**240**
North Macedonia	AG	0	0	0	0	0	0	0	1	0	0	2	0	0	5	0	0	0	**8**
	GEN	0	0	0	0	0	0	0	0	0	0	0	0	0	0	0	0	0	**0**
Norway	AG	0	0	0	0	0	0	0	0	0	0	0	0	0	0	0	0	0	**0**
	GEN	0	4	2	1	0	4	4	10	7	14	18	4	3	0	0	0	0	**71**
Russia	AG	0	3	1	4	9	13	16	9	16	12	2	0	0	0	0	0	0	**85**
	GEN	0	0	0	0	0	0	0	0	0	0	0	0	0	0	0	0	0	**0**
Spain	AG	0	0	0	0	0	0	0	0	0	0	0	0	0	0	0	0	0	**0**
	GEN	1	2	2	4	6	16	16	25	19	36	61	21	3	0	0	0	0	**212**
Sweden	AG	0	0	0	0	0	0	0	0	0	0	0	0	0	0	0	0	0	**0**
	GEN	2	4	5	11	13	10	8	12	8	7	12	11	8	2	0	0	0	**113**
Switzerland	AG	0	0	0	0	0	0	0	2	1	0	0	0	1	0	3	0	0	**7**
	GEN	0	0	0	0	0	0	0	0	0	0	0	0	0	0	0	0	0	**0**
United Kingdom	AG	0	1	0	0	0	0	0	1	1	2	0	0	2	1	0	0	0	**8**
	GEN	0	4	2	3	1	0	2	2	2	4	4	3	6	1	0	0	0	**34**
**Total**	**AG**	**1**	**5**	**1**	**5**	**10**	**13**	**16**	**15**	**18**	**15**	**6**	**5**	**7**	**14**	**7**	**5**	**1**	**144**
	**GEN**	**18**	**36**	**43**	**56**	**38**	**47**	**54**	**71**	**56**	**86**	**116**	**89**	**60**	**27**	**30**	**14**	**2**	**843**

AG: antigenic; GEN: genetic; TESSy: The European Surveillance System; WHO: World Health Organization.

^a^ The number of virus characterisations are as reported to TESSy (in both aggregated and strain-based reports).

Light blue and light green respectively indicate antigenic and genetic characterisations.

Reported antigenic data (Figure 2) are presented in Table 2. In their vast majority (134/144, 93%), the antigenic data were not linked with genetic data this season.

**Table 2:** Antigenic and genetic characteristics of influenza viruses as reported to TESSy^a^ by week of sampling, WHO European Region, week 40 2021–week 4 2022 (n = 987 characterisations)

Year Week	ANTIGENIC							TOTAL	GENETIC															TOTAL	
	AH1/ Guangdong-Maonan/ SWL1536/ 2019^b^	AH1/ Victoria/ 2570/ 2019^c,d,e^	AH3/ Cambodia/ e0826360/ 2020^d^	AH3/ Darwin/ 9/ 2021^e^	AH3 no cat	BVic/ Austria/ 1359417/ 2021^e^	BVic/ Brisbane/ 60/ 2008		AH1 subgroup not listed	AH3 subgroup not listed	BYam/ Phuket/ 3073/ 2013_ Clade 3^f^	BVic/ Washington/ 02/ 2019_ 1A.3^b,c,d^	AH1/ Guangdong-Maonan/ SWL1536/ 2019_ 6B.1A.5a.1^b^	AH1/ Victoria/ 2570/ 2019_ 6B.1A.5a.2 ^c,d,e^	AH1 no clade	AH3/ Denmark/ 3264/ 2019_ 3C.2a1b.1a	AH3 no clade	BVic no clade	BYam no clade	AH1/ India/ Pun-NIV312851/ 2021_ 6B.1A.5a.2	AH3/ Bangladesh/ 4005/ 2020_ 3C.2a1b.2a.2	AH3/ Cambodia/ e0826360/ 2020_ 3C.2a1b.2a.1 ^d^	BVic/ Austria/ 1359417/ 2021 _1A.3a.2 ^e^	
2021																									
**40**	0	0	1	0	0	0	0	1	0	0	0	0	0	0	0	0	0	0	0	0	18	0	0	**18**
**41**	0	0	5	0	0	0	0	5	0	0	3	0	0	0	0	1	0	0	0	0	32	0	0	**36**
**42**	0	0	1	0	0	0	0	1	0	0	0	0	0	1	1	0	0	0	2	0	39	0	0	**43**
**43**	0	0	4	1	0	0	0	5	0	0	1	1	1	1	1	0	1	0	1	1	48	0	0	**56**
**44**	0	0	8	1	0	1	0	10	0	0	0	0	1	0	0	0	0	1	0	3	33	0	0	**38**
**45**	0	0	12	1	0	0	0	13	0	0	0	1	1	0	0	0	0	0	0	1	43	0	1	**47**
**46**	0	0	15	1	0	0	0	16	0	0	0	0	0	0	0	1	3	0	0	0	49	1	0	**54**
**47**	0	0	11	2	1	0	1	15	0	0	0	0	0	0	0	0	3	0	0	0	67	0	1	**71**
**48**	0	0	11	6	0	1	0	18	0	0	0	0	1	0	0	1	2	0	0	0	52	0	0	**56**
**49**	1	0	6	8	0	0	0	15	1	0	0	0	3	0	1	0	12	0	0	0	69	0	0	**86**
**50**	0	0	3	1	2	0	0	6	2	0	0	0	2	0	0	1	6	0	0	0	104	0	1	**116**
**51**	1	0	4	0	0	0	0	5	1	0	0	0	5	0	1	2	20	0	0	0	58	0	2	**89**
**52**	0	0	6	1	0	0	0	7	0	0	0	0	5	0	0	1	2	0	0	0	52	0	0	**60**
2022																									
**1**	0	0	9	0	5	0	0	14	0	0	0	0	1	0	0	0	9	0	0	0	17	0	0	**27**
**2**	0	0	5	2	0	0	0	7	0	0	0	0	0	0	0	0	17	0	0	1	12	0	0	**30**
**3**	0	1	4	0	0	0	0	5	0	0	0	0	2	0	0	0	9	0	0	0	3	0	0	**14**
**4**	0	0	1	0	0	0	0	1	0	0	0	0	0	0	0	0	2	0	0	0	0	0	0	**2**
**ALL**	**2**	**1**	**106**	**24**	**8**	**2**	**1**	**144**	**4**	**0**	**4**	**2**	**22**	**2**	**4**	**7**	**86**	**1**	**3**	**6**	**696**	**1**	**5**	**843**

No cat.: no category; TESSy: The European Surveillance System; WHO: World Health Organization.

^a^ Antigenic and genetic characteristics of influenza viruses are shown in the Table as reported to TESSy (in both aggregated and strain-based reports) by week of sampling. When both record types are reported from same data source and week, only strain-based reports are considered.

^b^ WHO recommended vaccine viruses for the 2020/21 northern hemisphere influenza season (trivalent vaccines).

^c^ WHO recommended vaccine viruses for the 2021 southern hemisphere influenza season (trivalent vaccines).

^d^ WHO recommended vaccine viruses for the 2021/22 northern Hemisphere influenza season (trivalent vaccines).

^e^ WHO recommended vaccine viruses for the 2022 southern hemisphere influenza season (trivalent vaccines).

^f^ WHO recommended B/Yamagata-lineage vaccine virus for all four seasons (quadrivalent vaccines).

No cat.: not attributed to a predefined TESSy antigenic category. No clade: not attributed to a predefined TESSy genetic clade.

## Circulating influenza viruses at the beginning of the 2021/22 season in relation to northern hemisphere vaccine components

Because the antigenic data reported to TESSy were not linked to genetic data, they could not be used for assessing the apparent discrepancies of circulating clade proportions or the similarity of the circulating strains with the respective vaccine virus components. The WHO CC assessments for the 2022 NH VCM were used instead [7]. The characterisations further reported are until week 4 2022.

Of the 790 genetically characterised A(H3N2) viruses, 776 (98%), belonged to subclade 3C.2a1b.2a.2, and seven belonged to clade 3C.2a.1b.1a. Only one was subclade 3C.2a1b.2a.1, represented by A/Cambodia/e0826360/2020, the virus component for 2021/22 NH vaccines and the rest were not assigned to any predefined TESSy category. In the Netherlands, 15% characterised 3C.2a1b.2a.2 A(H3N2) viruses were reassortants, antigenically similar to 3C.2a1b.2a.2 viruses, carrying 3C.2a1b.2a.2 HA genes and seven 3C.2a1b.1a gene segments (personal communication with Ron Fouchier and Adam Meijer, April 2022). In the Region, the most common 3C.2a1b.2a.2 viruses were defined by HA1 amino-acid substitutions D53N, N96S and I192F (390/610, 64%) or D53G, D104G and K276R (170/610, 28%). Antigenic data showed that antisera raised against A/Cambodia/e0826360/2020-like viruses, reacted with the 3C.2a1b.2a.2 viruses less well [7], but still retained a low level of recognition. At the 2022 NH VCM the recommendation was to change the A(H3N2) vaccine component for the NH 2022/23 influenza season to include a 3C.2a1b.2a.2 virus [1].

Of the 38 A(H1N1)pdm09 viruses assigned to a clade, 24 belonged to the 6B.1A.5a.1 and eight (represented by A/India/Pun-NIV312851/2021) to the 6B.1A.5a.2 subclade. The rest were not assigned to any predefined TESSy category. Two of the 38 viruses were represented by A/Victoria/2570/2019 (6B.1A.5a.2), the virus component for 2021/22 NH vaccines. Subclade dominance varied between countries. The most common 6B.1A.5a.1 subclades were defined by HA1 amino-acid substitutions S326P (3 sequences), P137S, G155E, S128T (3 sequences) and P137S, G155E, R113K, I298V (2 sequences). Antigenic data showed that 6B.1A.5a.1 viruses were less well inhibited by antisera raised against 6B.1A.5a.2 clade viruses [7]. At the 2022 NH VCM the recommendation was to retain A/Victoria/2570/2019 as the vaccine component taking into consideration the global perspective and human serology studies on pre- and post-influenza vaccinated subjects [1].

Of the 15 B viruses genetically characterised, eight were assigned to the B/Victoria lineage. Five of these belonged to clade V1A.3a.2, represented by B/Austria/1359417/2021, and only two were assigned to clade V1A.3, represented by B/Washington/02/2019, the recommended vaccine virus strain for the 2021/22 NH influenza season. The other B/Victoria virus was not assigned to any predefined TESSy category. Currently circulating V1A.3a.2 viruses carried HA1 A127T, P144L and K203R amino-acid substitutions. Antigenic data showed these viruses were poorly inhibited by post-infection ferret antisera raised against B/Washington/02/2019-like viruses [7]. At the NH VCM, the recommendation was to change the B/Victoria-lineage vaccine component to include B/Austria/1359417/2021-like (V.1A.3a.2) viruses [1]. All seven influenza B viruses that were genotyped as B/Yamagata-lineage were reported from Scotland. These reports coincided with the live attenuated influenza vaccine (LAIV) vaccination campaign in the UK. No sequence data were reported from those viruses and the laboratory could not exclude the possibility that these viruses originated from LAIV. There has been no confirmation of circulation of B/Yamagata lineage viruses, in the global context, since 2020. However, given the low numbers of type B viruses detected in the course of the 2021/22 season, and the small percentage that were genotyped, it was recommended at the NH VCM to retain B/Phuket/3073/2013-like(Y3) viruses for use in quadrivalent 2022/23 influenza vaccines [1].

## Discussion

Influenza is still circulating above the epidemic threshold in the WHO European Region as at week 10 2022 [9]. While we are in the third year of the COVID-19 pandemic, the public health emergency measures in place have continued to impact the circulation of influenza viruses in 2021/22, although less visibly than in the 2020/21 and 2019/20 seasons. With the lifting of public health measures, influenza detections rose again above the epidemic threshold in week 8 2022, and in the coming weeks influenza viruses may continue to co-circulate with SARS-CoV-2 and other respiratory viruses. Out-of-season influenza outbreaks cannot be excluded either, particularly with increasing international travel.

The need to prioritise resources for SARS-CoV-2 surveillance during the pandemic likely affected the proportion of influenza viruses that were characterised and their surveillance generally. As a result, the number of countries that reported influenza characterisation data were substantially fewer than in seasons before the COVID-19 pandemic.

For the 2021/22 influenza season up to week 10, A(H3) viruses have dominated in the Region and the majority (98%) of characterised viruses up to week 4 2022 fell in genetic clade 3C.2a1b.2a.2, being distinct from the NH vaccine component for the 2021/22 season vaccines. This may have negative implications for the vaccine effectiveness, as has been described by the United States interim estimates, whereby seasonal influenza vaccination did not reduce the risk for outpatient respiratory illness caused by influenza A(H3N2) viruses that have predominated up to mid-February 2022 [12]. Of the few A(H1N1)pdm09 and B/Victoria-lineage viruses detected, most have fallen in clades 6B.1A.5a.1 and V1A.3a.2, respectively, and show reduced recognition by post-infection ferret antisera induced by the 2021/22 vaccine component viruses, which were clade 6B.1A.5a.2 and V1A.3 viruses.

The NH 2022/23 season vaccine recommendations reflect the observed antigenic drift of A(H3N2) and B/Victoria lineage viruses, with the respective components changed to include 3C.2a1b.2a.2 and V1A.3a.2 clade viruses. However, the 6B.1A.5a.2 A(H1N1)pdm09 component remained unchanged [7,13]. While vaccination remains the best way to protect against influenza and severe disease, due to the potentially reduced VE in 2021/22 season against the widely circulating A(H3N2) viruses, antivirals (oseltamivir, zanamivir and baloxavir) should also be used to manage influenza infection in high-risk and elderly groups irrespective of their vaccination status. All viruses tested during 2021/22 remain sensitive to these antivirals [9].

While there have been no confirmed reports of B/Yamagata-lineage circulation since March 2020 [13-15], laboratories need to enhance surveillance of influenza B viruses and genotyping of these viruses to determine their lineage. This need is highlighted by the fact that most detected influenza B viruses this season so far (99%, 1,581/1,603 up to week 10 2022) remained lineage undetermined. Such data, with detailed characterisation of any B/Yamagata-lineage viruses detected, would be important to facilitate evidence-based decisions at forthcoming influenza VCMs regarding the possible disappearance of B/Yamagata lineage viruses and future decisions on quadrivalent influenza vaccines. In the absence of circulating B/Yamagata-lineage viruses, there should be awareness of the possibility of B/Yamagata-lineage detections originating from the vaccine during the LAIV vaccination period.

Our study has some limitations. Characterisation data up to week 4 2022 have been considered and therefore it cannot be excluded that the proportions of circulating influenza types and subtypes may change and that different clades and subclades spread throughout the remainder of the season. As only 22% (11/50) of the countries that report surveillance data to TESSy reported also genetic data, the proportion of different clades may not be generalisable to the WHO European Region as a whole. 

## Conclusion

NICs play a crucial role in surveillance of influenza being responsible for providing representative specimens to the WHO CC for making VCM recommendations. As influenza presently continues to circulate, NICs should keep up testing and characterising viruses throughout the remainder of current season, while staying vigilant to detect out of season influenza outbreaks as well. Characterisation data from the NH will inform the VCM decisions in the SH VCM that takes place in September─October 2022. The transition towards surveillance systems that integrate influenza, SARS-CoV-2 and other respiratory pathogens may pose challenges to the surveillance systems that need to collect, analyse and timely report data. During this transition period, laboratories will need to ensure that representative specimens for influenza virus detection continue to be collected, and subsequent virus characterisations are performed and reported [10,11].

## Supplementary Data

123000 Supplement S1
