# Supplementary material for: Influenza returns with a season dominated by clade 3C.2a1b.2a.2 A(H3N2) viruses, WHO European Region, 2021/22
Source: Euro Surveill. 2022 Apr 14;27(15):2200255. doi: 10.2807/1560-7917.ES.2022.27.15.2200255 (PMC9012087; doi:10.2807/1560-7917.ES.2022.27.15.2200255)
Supplement: Supplement S1 [file 2200255_Supplement1.pdf]

This supplementary material is hosted by *Eurosurveillance* as supporting information alongside the article ‘Influenza returns with a season dominated by clade 3C.2a1b.2a.2 A(H3N2) viruses, WHO European Region, 2021/22’ on behalf of the authors who remain responsible for the accuracy and appropriateness of the content. The same standards for ethics, copyright, attributions and permissions as for the article apply. Supplements are not edited by Eurosurveillance and the journal is not responsible for the maintenance of any links or email addresses provided therein.

S1. Influenza Season in the WHO European Region: weeks 40/2021-10/2022, 40/2020-10/2021, 40/2019-10/2020

Virus type/subtype/lineage	Cumulative number of detections for weeks 40/2021-10/2022			Totals*		Cumulative number of detections for weeks 40/2020-10/2021			Totals*		Cumulative number of detections for weeks 40/2019-10/2020			Totals*	
	Sentinel sources	Non-sentinel sources	Totals	%	Ratios	Sentinel sources	Non-sentinel sources	Totals	%	Ratios	Sentinel sources	Non-sentinel sources	Totals	%	Ratios
**Influenza A**	**3045**	**50401**	**53446**	**97.1**	**33.3:1**	**21**	**347**	**368**	**50.6**	**1:1**	**10671**	**99729**	**110400**	**74.3**	**2.9:1**
A(H1N1)pdm09	173	1232	1405	7.6		13	28	41	48.2		5839	17606	23445	55.1	
A(H3N2)	2094	15002	17096	92.4	12.2:1	6	38	44	51.8	1.1:1	3933	15154	19087	44.9	0.8:1
A not subtyped	778	34167	34945			2	281	283			899	66969	67868		
**Influenza B**	**38**	**1565**	**1603**	**2.9**		**14**	**345**	**359**	**49.4**		**5709**	**32423**	**38132**	**25.7**	
Victoria lineage	6	16	22	100.0		1	7	8	80.0	4:1	2142	1708	3850	98.0	48.1:1
Yamagata lineage	0	0	0			0	2	2	20.0		21	59	80	2.0	
Lineage not ascribed	32	1549	1581			13	341	354			3546	30656	34202		
**Total detections**	**3 083**	**51 966**	**55 049**			**35**	**692**	**727**			**16 380**	**132 152**	**148 532**		
**Total tested**	**39 551**	**>1 849 356**	**>1 888 907**			**27 779**	**>454 125**	**>481 904**			**44 090**	**>587 319**	**>631 409**		
**Sentinel % positive**	**7.8**					**0.1**					**37.2**				
**% Total detections**	**5.6**	**94.4**				**4.8**	**95.2**				**11.0**	**89.0**			

[a] Numbers taken from Flu News Europe to week 10/2022, week 10/2021 and 10/2020 reports for the three influenza seasons

* Percentages are shown for total detections (types A & B [in bold type], and for viruses ascribed to influenza A subtype and influenza B lineage). Ratios are given for type A:B [in bold type], A(H3N2):A(H1N1)pdm09 and Victoria:Yamagata lineages.
